# Pathological Diagnosis, Work-Up and Reporting of Breast Cancer 1st Central-Eastern European Professional Consensus Statement on Breast Cancer

**DOI:** 10.3389/pore.2022.1610373

**Published:** 2022-06-27

**Authors:** Gábor Cserni, Monika Francz, Balázs Járay, Endre Kálmán, Ilona Kovács, Tibor Krenács, Erika Tóth, Nóra Udvarhelyi, László Vass, András Vörös, Ana Krivokuca, Karol Kajo, Katarína Kajová Macháleková, Janina Kulka

**Affiliations:** ^1^ Department of Pathology, Bács-Kiskun County Teaching Hospital, Kecskemét, Hungary; ^2^ Institute of Pathology, University of Szeged, Szeged, Hungary; ^3^ Department of Pathology, Szabolcs-Szatmár-Bereg County Hospitals and University Teaching Hospital, “Jósa András” Teaching Hospital, Nyíregyháza, Hungary; ^4^ Medserv Kft., Budapest, Hungary; ^5^ Institute of Pathology, University of Pécs, Pécs, Hungary; ^6^ Department of Pathology, University of Debrecen, “Kenézy Gyula” University Hospital, Debrecen, Hungary; ^7^ Department of Pathology and Experimental Cancer Research, Semmelweis University, Budapest, Hungary; ^8^ Department of Pathology, National Institute of Oncology, Budapest, Hungary; ^9^ Department of Pathology, Pest County “Flór Ferenc” University Teaching Hospital, Kistarcsa, Hungary; ^10^ Institute for Oncology and Radiology of Serbia, Belgrade, Serbia; ^11^ Department of Pathology, St. Elisabeth Cancer Institute, Bratislava, Slovakia; ^12^ Department of Pathology, Forensic and Insurance Medicine, Semmelweis University, Budapest, Hungary

**Keywords:** pathology, breast cancer, diagnostics, consensus conference, recommendations

## Abstract

This text is based on the recommendations accepted by the 4th Hungarian Consensus Conference on Breast Cancer, modified on the basis of the international consultation and conference within the frames of the Central-Eastern European Academy of Oncology. The recommendations cover non-operative, intraoperative and postoperative diagnostics, determination of prognostic and predictive markers and the content of cytology and histology reports. Furthermore, they address some specific issues such as the current status of multigene molecular markers, the role of pathologists in clinical trials and prerequisites for their involvement, and some remarks about the future.

## Introduction

The pathology panel of the 1st Central–Eastern European Professional Consensus Statement on Breast Cancer has based its recommendations principally on the consensus document on breast cancer diagnosis, work-up and reporting achieved at the recent 4th Hungarian Breast Cancer Consensus Conference, which itself was based on previously published national and international recommendations (1–14), of which the newest ones are dealt with in subsequent parts of this document. The original source text took into account the legitimate demands of allied disciplines and the possibilities of pathologists, and changes were made to the text, where deemed necessary as a result of developments since the acceptance of the source document or consultations of the international panel of the Eastern European Professional Consensus Statement on Breast Cancer. The recommendations formulated in this document provide a possible diagnostic, processing and reporting guideline that may help in the optimal detection and management of breast diseases. The professional panel considers that its guidance should be followed, provided that personal and material conditions are met. The evidence behind these recommendations, apart from those specifically indicated, is mostly of the lowest level and reflect expert consensus, as this is a diagnostic area that has generally not (or only to a limited extent) been validated by clinical trials.

In the diagnosis of breast diseases, non-operative/preoperative diagnostics have become a key starting point for the treatment of patients. Diagnosis obtained intraoperatively has lost its previous significance; it is now accepted that diagnostic steps should be undertaken in all cases to establish the diagnosis before surgery/treatment.

## Non-Operative Diagnostics (Preoperative or Pretreatment Biopsy Diagnosis)

Non-operative/preoperative pathological diagnostics is part of the “diagnostic triad” (clinical examination, radiology, pathology). It is important for the pathologist to know the results of other investigations, and to take these into account when giving an opinion on the case. If the pathological diagnosis is made in an isolated setting, without knowledge of clinical and radiological context, this can be a source of serious mistakes and errors. As a minimum requirement for pathological specimens, the localization of the lesion, findings from the physical examination, radiomorphology of the lesion, the radiologist’s opinion on the lesion, the method of sampling, and the relevant data in the medical history (e.g., history of malignancy of other organs, pregnancy/lactation at the time of sampling) should be included in the request form. In an optimal situation, the pathological findings, together with the results of other investigations, are placed in an appropriate diagnostic/therapeutic context within a multidisciplinary framework. If all findings are consistent, an appropriate therapeutic decision can be taken, while in the event of inconsistency, further diagnostic steps should be implemented.

It should be noted that, like all diagnostic tests, non-operative diagnostics have limitations. These limitations are reflected by the proportions of “acceptable” false negatives, false positives, non-evaluable and “suspicious” cases specified in the European Guidelines (Table 1) (6).

**TABLE 1:** Recommended minimum values for selected quality characteristics, based on European directives (6).

Cytology	Minimum	Recommended
Positive predictive value (PPV)	>98%	>99%
False negative rate (FNR)	<6%	<4%
False positive rate (FPR)	<1%	<0.5%
Inadequate rate (INAD)	<25%	<15
Inadequate rate for cancers	<10%	<5%
Suspicious rate	<20%	<15%
**Core biopsy**		
Positive predictive value (PPV)	>99%	>99.5%
False negative rate (FNR)	<0.5%	<0.1%
(B1+B2) ratio for cancers	<15%	<10%
Suspicious rate	<10%	<5%

Pathological (cytological or histological) evaluation of a radiologically or clinically detected lesion raising the slightest suspicion of malignancy is always justified for clarification of the lesion; exceptions to this are very rare. For lesions considered benign, confirmation of benignity may also be a goal. Non-operative diagnosis may be established using a sample obtained by guided fine-needle aspiration or core needle biopsy with an automated gun or possibly with a vacuum-assisted biopsy device. For fine-needle aspiration, we recommend the use of European (UK) terminology (6, 13) or the more recent Yokohama terminology (15–17). In essence, the latter does not differ from the earlier European diagnostic category recommendations; rather, these are supplemented with a percentage risk of malignancy (ROM) associated with each category. It is also recommended to supplement the diagnostic categories with the C1−C5 categories, which are easier to use for statistical purposes (e.g., to calculate absolute and complete specificity, or sensitivity of biopsy samples) and which are still not recommended to be used alone.

For core needle biopsies, the B1−B5 category classification is a requirement (Table 2) (6, 13, 15, 17), but these categories also cannot stand alone without a written opinion. Efforts should also be made to provide additional information, such as diagnosis, limited prognostic information, histological type for cancers, nuclear or estimated histological grade, prognostic and predictive factors for planned neoadjuvant/primary systemic therapy (PST); see below.

**TABLE 2:** Definition of non-operative diagnostic categories.

Cytological diagnostic categories	
United Kingdom/European Recommendation (6,13)	Recommendation of the International Academy of Cytology, Yokohama (15–17) (Risk of malignancy: ROM%)
C1: Inadequate (quantitatively and/or qualitatively)	Inadequate (2.4–4.58%)
C2: Benign lesion	Benign (1.2–2.3%)
C3: Atypical, probably benign	Atypical (probably benign) (13–15.7%)
C4: Suspicious of malignancy	Suspicious (of malignancy) (87.6–97.1%)
C5: Malignant (both *in situ* and invasive)	Malignant (99–100%)
**Core biopsy categories (6)**	
B1: Normal breast tissue/Uninterpretable	
B2: Benign lesion	
B3: A lesion with uncertain malignant potential (malignancy may be associated with ≤25% of cases in the group as a whole).	
The followings are typically included in this category	
– Some sclerosing lesions: radial scars, complex sclerosing lesions, sclerosing papillomas	
– Non-malignant papillary lesions that have not been completely removed	
– Lobular (intraepithelial) neoplasia (atypical lobular hyperplasia, classical LCIS; cf. B5a)	
– Atypical epithelial proliferation of ductal type (this name is recommended for atypical epithelial proliferation of ductal type found in core biopsies, as quantitative criteria for atypical ductal hyperplasia (ADH) cannot be evaluated in core biopsy samples, so the diagnosis of ADH is not possible on core biopsy)	
– Mucocele-like lesions	
– Cellular fibroepithelial lesions	
– Spindle cell lesions for which other classification is not possible based on the sample	
B4: Suspicious of malignancy	
B5: Malignant	
B5a: *in situ* carcinoma ( ductal carcinoma *in situ*, pleomorphic and florid lobular carcinoma *in situ*; compare with B3; note: the United Kingdom recommendation for florid lobular carcinoma *in situ* is B4)	
B5b: invasive breast carcinoma	
B5c: indeterminate, either an *in situ* or an invasive carcinoma	
B5d: other malignant process	

Categories C2, B2 (benign) and C5, B5 (malignant) can be considered definitive diagnoses, but these should be interpreted only in a multidisciplinary environment together with imaging and clinical findings, in a “triple diagnostic system”. Diagnostic categories should not be used without a written opinion. Categories are primarily useful for statistical evaluation purposes and assist in patient management.

The use of (mainly ultrasound-) guided sampling is recommended even for palpable lessions, due to the possible differences between the palpated and the actual size of the lump or possible necrosis. With the use of image-guidance, it is also easier to establish that there is no other circumscribed lesion responsible for the palpatory finding (e.g., fat lobule), or that the palpatory finding does not match the lesion found on diagnostic imaging.

Calcifications that are suspicious for malignancy should be evaluated primarily using core needle biopsy or vacuum-assisted core biopsy. If, for some reason, such calcifications are still sampled by fine-needle aspiration, a negative result is not sufficient to rule out malignancy; the result of aspiration cytology is only acceptable if it confirms the suspicion of malignancy. Core biopsies have also become relevant in other clinical situations and should be preferred to cytology sampling; if a biomarker assay is likely to be performed when considering or planning PST, it can be performed more reliably on core needle biopsy samples than on cytological specimens (18).

Since atypical ductal epithelial proliferations and DCIS (ductal carcinoma *in situ*) may form a spatial spectrum of lesions, a core needle biopsy taken from the area of microcalcification will not necessarily be representative. The situation may be similar for B3 category papillary and sclerosing lesions. Therefore, excision may be required for a reliable diagnosis of these lesions. A multidisciplinary approach to B3 entities has also resulted in an international consensus agreement to avoid over-treatment and under-diagnosis. In a more recent recommendation, among lesions classified B3, diagnostic excision may be avoided in papillary and radial sclerosing lesions. If a vacuum-assisted biopsy is performed and the sample is large enough, a papillary lesion may also be considered a papilloma (B2), and this type of biopsy is sometimes suitable for removing the entire lesion visualized radiologically, and subsequent surgery will not be necessary (19). Establishing and documenting radiopathological correlation and team-based decision-making is mandatory for B3 lesions, especially for vacuum-assisted excisions.

When planning a primary systemic (neoadjuvant) treatment, high-quality core needle biopsy material from the primary tumour should be preferred (exceptionally, incisional biopsy may be acceptable), and in each case, predictive factors should also be determined (as a minimum, oestrogen and progesterone receptor and HER2 status should be assessed, and, if requested, a marker to characterize proliferation, usually the Ki67 labelling index and the proportion of stromal tumour infiltrating lymphocytes (sTIL): see below for details). According to international (European Society for Medical Oncology) recommendations, a core biopsy with several (at least 2–3) tumour tissue cylinders is the expectation in such cases (20). When assessing the effects of therapy, a comparison of the histological picture of the tumour in the core needle biopsy and after neoadjuvant treatment is also an internationally recommended requirement (12).

It is a generally accepted view that mastectomy cannot be performed based solely on cytological opinion, but this may be acceptable in exceptional cases involving reliable, well-synchronized teams. If the cytological and radiological opinions differ markedly, (e.g., C2/R4-5 or U 4/5 or C4-5/R1-2 and U1-2), repeated sampling and core needle biopsy should always be considered.

Efforts should be made to evaluate both histological and cytological specimens in reliable, quality-assured laboratories. Departments are expected to participate in external quality control programmes and meet compliance requirements. Pathology reporting of breast samples also requires sufficient skills, for which there are no defined criteria in most countries, but an international recommendation (EUSOMA: European Society of Breast Cancer Specialists) sets the minimum workload required for proficiency at 50 cases of early breast cancer surgical specimens, prefereably 100 (but at least 50) non-operative/preoperative samples and 25 metastatic cases per year (21). Secondary certification exams (e.g., cytology) might also be a requirement for recognizing proficiency in countries where such graduation exists.

Non-operative diagnosis of lymph node status will be discussed in the section on lymph nodes.

### Processing Core Biopsies

It is essential that the tissue cylinders are placed into the block parallel to their longitudinal axis. Usually 2–3 cylinders, 1 mm in thickness and 10 mm in length are obtained for assessment. [The number of cores (tissue cylinders) will determine how representative the biopsy is and is proportional with the likelihood of establishing a correct diagnosis (22)]. These are examined by following the rules for small biopsies and if needed, multiple layers are obtained. It may be advisable to place serial tissue sections immediately on pretreated slides since the area in question may be cut out before immunohistochemistry is performed. Haematoxylin-eosin (HE) stained sections placed on treated slides are also suitable for performing immunohistochemical reactions in a second step. For a core biopsy (or other small-volume biopsies), it may be necessary to prepare a relatively large number of sections in several rounds, which leads to significant material loss due to multiple trimmings and sectionings. In such cases, the sample should be further examined after dividing it into multiple parts (e.g., if tissue cores were embedded into a single block, they should be reembedded into separate cassettes, or longer cores should be halved). This may be needed since PST may result in complete or nearly complete regression, and when a new tissue-based predictive test is required in such cases, the remaining core biopsy of the primary tumour may be the most readily available sample. Providing a core biopsy tumour sample may also be an inclusion criterion for participation in clinical trials. Quantitative characterization of the relevant lesion present in the core biopsy is also recommended [for example, in addition to the nature of the pathological abnormality responsible for microcalcification—e.g., columnar changes, flat epithelial atypia (FEA), atypical epithelial hyperplasia—the percentage or length in mm can be given].

From core biopsy samples obtained before neoadjuvant treatment, tumour characteristics influencing the treatment should be determined, and in addition to predictive factors, the following should also be described, if possible: vascular invasion and presence of an *in situ* component; more recently, neoadjuvant treatment may require quantification of stromal tumour infiltrating lymphocytes (sTILs) (23).

Another diagnostic modality of biopsy is vacuum-assisted biopsy (VAB; vacuum mammotomy), which is performed with a 7G to 11G needle under ultrasound (US), stereotaxis or magnetic resonance imaging (MRI) guidance. It is a minimally invasive breast biopsy that removes more tissue than traditional gun CNBs, allowing the removal of smaller lesions, making VAB a therapeutic alternative for some lesions (19). For vacuum-assisted biopsies, larger volume samples are processed, in the form of tissue cylinders or smaller fragments, depending on the device. If cylinders containing calcification have been separated by the sampler, it is advisable to process them separately during histological examination. If necessary, decalcification using EDTA (ethylenediamine tetraacetic acid) is recommended; the use of strong acids should be avoided (24).

For tissue biopsies taken from microcalcifications, it is advisable to indicate the approximate size of calcifications on microscopic examination since small calcifications (below 50 μm) are unlikely to be detected on mammography, unless multiple similar foci are superimposed; thus, stating the size of calcifications helps to establish a proper radiopathological correlation. If the core biopsy/vacuum-assisted core biopsy was performed because of microcalcification, specimen radiography of the sample is a requirement (this will validate sampling) and, optimally, calcified particles may also be sent separately for analysis. If microcalcificates do not appear in the first sections, deeper sections will be required. If microcalcifications cannot be confirmed by routine microscopic evaluation, polarized light may be helpful, since calcium oxalate crystals (weddellite) are refractile and polarizable but usually clear or tinged yellow in H&E sections (25).

Exceptionally (e.g., after multiple unsuccessful cytological or core biopsy samplings of a large, radiologically suspicious lesion; for extensively ulcerated, advanced breast tumours; in Paget’s disease; for very superficial lesions), a minimally invasive surgical intervention may also serve as a preoperative diagnostic method (incisional biopsy).

## Intraoperative Examinations

• Intraoperative examinations may be macroscopic examinations with the naked eye or microscopic examinations (analysis of imprint or scrape cytology samples or frozen sections). All of these have limitations compared to permanent section histology; it should be highlighted that the quality and evaluability of frozen sections is poorer than that of permanent sections. Intraoperative molecular tests are not performed in most central—eastern European countries. There are also examples of intraoperative immunohistochemistry in the literature, with both imprint cytology and frozen section variants increasing the sensitivity of lymph node examination; however, these generally reveal only small metastases that would not affect the outcome of surgery, therefore routine intraoperative immunohistochemistry is not justified.
• For large lesions found to be *in situ* carcinomas on radiological and/or preoperative pathology examinations, and for lesions detected exclusively in the form of microcalcifications, intraoperative frozen section examination is meaningless because it does not help to clarify the diagnosis and may render the tissues unsuitable for making the eventual diagnosis. For this reason, no frozen section exam is performed on such samples.
• Frozen sections must not be prepared from lesions of 10 mm or less, since failure to obtain a sufficient quantity and quality of tissue from the lesion for embedding will jeopardize definitive diagnosis and also the ability to assess prognostic and predictive factors for small invasive tumours. If there is a definitive preoperative diagnosis, there is no need for intraoperative examination to confirm this diagnosis. Frozen sections should not be used merely to compensate for inadequate preoperative evaluation.
• The indications for frozen section examination have become significantly limited. In exceptional cases, if attempts to obtain a preoperative diagnosis have failed, a multidisciplinary decision may be made to examine frozen sections; this may also be justified if there are insufficient or uncertain preoperative findings, in similarly very rare instances.
• The aim of intraoperative examination may also be the assessment of surgical resection margins or the distance between the tumour and the tumour-free margin. These examinations can be performed as imprints (cytology), frozen sections and macroscopic measurements. (In the latter cases, the original resection surface must be marked with dye before incision!)
• Intraoperative examinations may also be done to assess sentinel lymph node status.
• The final decision on the nature and feasibility of an intraoperative examination is made by the pathologist.
• Molecular tests, tissue banking: If the infrastructure allowing tissue samples to be frozen and stored at −80°C is available, it is recommended that a part of the tumour tissue be stored in this manner after proper orientation of the freshly resected tissue and marking of surgical surfaces (see below). Of course, tissue banking can be inititated only if this does not reduce the diagnostic possibilities; the priority should be for making the proper diagnosis and for assessing parameters influencing treatment. A key point of whole tissue biobanking is the time factor of the ischemia of the harvested tissue. According to several studies, it is recommended that the material be collected for freezing within 15–30 min after the interruption of the blood supply in order to minimize the hypoxic damage. If the specified time of ischemia is exceeded, irreversible processes could occur at the molecular level, which would impair the quality of biomolecules. As the time interval between surgical resection and freezing of the tissue is relatively short, biobanking requires a perfect interaction and cooperation of the workplaces involved, as well as experienced and trained pathologists.

## Postoperative Diagnostics—Processing, Principles of Cooperation

• Surgical materials should be sent for pathological examination accompanied by clinical data described for non-operative diagnostics. If neoadjuvant treatment has been administered, it is essential to state this, indicating original tumour size, location, tumour data obtained from a biopsy specimen taken prior to treatment, nature of the treatment, and the clinically evaluated response to treatment. The pathologist should be informed of the type of surgery. Surgical resections (breast operations) are divided into breast conserving procedures (inclusive of excision, segmental resection, lumpectomy, quandrantectomy, segmental/sectoral or partial mastectomy … etc., with or without axillary surgery and different methods of oncoplastic surgery) and total mastectomy (simple, skin-sparing, nipple-sparing, modified radical and radical mastectomy).
• The surgical specimen should be made available to the pathology department/pathologist immediately after removal (within a maximum of 30–60 min), without fixation and incision. If this is not feasible, the guidelines for sample fixation described under the section on “Special assessment of prognostic and predictive factors” are to be followed. Correct processing generally requires a preoperative mammography and specimen mammography image annotated by the radiologist and the related radiology report to be available to the pathologist at the time of the cut-up. This is essential for most breast-conserving surgeries, multifocal tumours, extensive DCIS, and surgical preparations following primary systemic treatment. It is recommended that macro-photography and/or a simple drawing be done of the slices, especially for small lesions, and that a specimen mammographic image of the slices be captured, especially for lesions with microcalcifications.
• The multifocal character of the lesion is determined primarily by the radiologist and secondarily by the pathologist. Instead of conventional classification of tumours with multiple foci (multifocal or multicentric), it is advisable to mention a certain number of focal lesions or multiple tumours/tumours with multiple foci. pT classification is made based on the largest focus, with indication of multifocality, since this is associated with a worse prognosis (26–28). Besides pT classification, it is also advisable to specify the extent of the tumour, which is the distance between the most distant margins of the two most distant foci, i.e., the largest dimension of the breast parenchyma affected by the tumour. This may play a role in the planning of customized oncological therapy.
• As with all measurements, both macroscopic and microscopic assessment of tumour size is approximate, but it is essential that this be recorded. At a minimum, the greatest dimension of the tumour should be given. (This may fall into a different plane than the plane of slicing, therefore requiring the assessment of tumour size in all three dimensions.) If there is a discrepancy between macroscopic and microscopic measurement, the latter shall prevail, unless the tumour is so large that it is impossible or meaningless to measure it microscopically.
• Regardless of its size, the tumour should be processed in a representative manner, ideally achieved by examining the entirety of the cut surfaces in multiple planes. For large tumours, a minimum of 1 block/1 cm is recommended.
• The surgical specimen should be marked in the operating room, ideally *in situ* (e.g., with surgical stiches) (with at least three clear, ideally radiopaque markers, such as medial, lateral, superior pole; or central/mammillary, peripheral and clockwise; or with insertion of two sutures and specifying the side) for a proper orientation. The fact of orientation should also be recorded by the pathologist. It is recommended that the surgeon marks the fascia (e.g., with 4 clearly identifiable sutures placed at its borders) and that both the surgeon and pathologist make a statement about its presence. In nipple-sparing surgeries, identification of the retromammillary region is essential, and this should therefore also be labelled by the surgeon. The size of the surgical specimen is specified in cm in three dimensions, and its weight is also given, since this is the simplest and best way to characterise the volume, and can be used as a basis for assessment of certain surgical quality indicators.
• To allow the assessment of the resection margins, staining the resection surfaces of the surgical specimen is essential: most simply with one colour, but with at least two different colours (e.g., black—anterior surface, blue—posterior surface) to facilitate subsequent orientation, and ideally with 6 colours. Our understanding of the recommended minimum tumour-free margin has changed significantly recently. For early invasive breast cancer (stage I and II), on the basis of consensus based on results from randomized trials and meta-analysis (highest level of evidence), a margin is considered positive (i.e., justifying re-excision) when dye is seen on tumour cells (invasive or *in situ* component)—“ink on tumour” (29, 30). On the one hand, it should be emphasized that evidence for this recommendation does not apply to pure *in situ* carcinoma, patients receiving PST or tumours in patients who have undergone accelerated partial breast irradiation (APBI) (31), while on the other hand, we should be aware of the technical limitations which as a consequence may mean that the presence of dye does not necessarily indicate a resection surface (e.g., in case of artificial cracks in the adipose tissue, dye may seep into deeper layers; for tissues removed in multiple fragments, the relationship between them becomes uncertain). We should also be aware that—based on individual considerations—re-excision may be reasonable even in the absence of a tumour-positive margin, when phenomena associated with a higher risk for residual tumour (large tumour volume in the immediate vicinity of the margin, discontinuous growth pattern such as an extensive intraductal component, lobular histological type or diffuse infiltration) are present. For purely *in situ* tumours, a similarly high level of evidence for assessment of positive margins is not available. For DCIS, an international panel recommends a tumour-free margin of 2 mm (29), while for classical lobular neoplasia (LN), a tumour-positive margin does not imply any further therapeutic indication. (Since its introduction by Haagensen, LN is an umbrella term for atypical lobular hyperplasia and *in situ* lobular carcinoma, not including invasive tumours; however, it may be sometimes qualified by additional adjectives: e.g., non-invasive LN—see below under histological types). For pleomorphic and/or florid lobular neoplasia (pLCIS, fLCIS), there is no high-level evidence overriding previous treatment recommendations, which are similar to those relating to DCIS. Retrospective studies have shown that a pLCIS/fLCIS in the resection margin is associated with invasive lobular carcinoma in a sufficiently high proportion of cases to represent an additional treatment indication (31). For margin assessment in the multidisciplinary setting, an important additional information in the description of the surgical operative procedure may be whether the excision toward the chest has reached the fascia (or not). Taking color digital pictures made during the cut-up of the surgical specimen (including both the original specimen and the inked slices) and correlating them with scanned (digitized) histological slides helps to demonstrate the localisation of the positive surgical margins during the multidisciplinary discussion.
• It should be clearly identified whether there are one or more abnormal masses in the parenchyma.
• Blocks are sequentially numbered so that the location of each block within the original preparation can be accurately traced back based on the macroscopic description.
• All areas that appear abnormal, all parenchyma fragments containing microcalcification, are sampled in a sufficiently representative manner. If mammography images or macro photos of the slices have been captured, it is advisable to indicate the location of blocks on the film/digital image or on a schematic drawing. A schematic drawing that also reflects orientation often carries more information than a block list and lengthy descriptions, which may be expressed in local jargon. For this reason, it is important to have this visual information to hand during reporting, and (for example) if an external consultation is requested, a copy of these drawings (block maps) should also be sent to the consulting professional.
• Besides sampling from the tumour for histological examination, it is also essential to sample apparently intact areas around the tumour, including surgical resection surfaces.
• If a marker clip has been inserted, its documentation (its absence or presence on specimen mammography) is part of the pathological assessment.
• The remaining slices of the specimen are to be kept in order and stored in a way that best enables reconstruction (e.g., wrapped in gauze).
• Re-excision is required if excision was not performed with negative margins; the specimen from the re-excision should also be oriented, primarily in order to establish the relationship with the previous excision. This is the only way to perform the pathological evaluation of the new resection surfaces.
• When there is a discrepancy between a clinical diagnosis and the diagnosis of the surgical material, a comparison with a preoperative biopsy specimen may resolve this contradiction; therefore, if preoperative assessment was performed at another institution, it is recommended that the pathological specimen be requested and reexamined.
• If uniform orientation principles are adhered to, there are few cases in which, due to uncertainty, it may be necessary for the surgeon to review the surgical material before slicing, but in such cases, it is inappropriate to omit this step. Postoperative discussions provide an excellent opportunity for verifying that the screen-detected and removed tumour was identical.
• In the vast majority of cases, intraoperative specimen mammography is performed in the radiology department that previously diagnosed the lesion. Pathology departments may also perform this examination if they are properly equipped, but the captured image should always be compared with the original mammogram. During pathological processing, the presence of the original mammographic image and comparison with specimen mammography are also important. If the pathologist has any issues with the interpretation of the specimen mammogram, consultation between the two professions is warranted. Optimally, a joint evaluation in person should be carried out; this is not always possible, but it can be replaced by various alternative solutions (e.g., consultation *via* remote communications). If an MRI has also been performed, preferably the MRI report and the visual material of the scanning should be made available, along with the possibility of consultation with a radiologist experienced in breast diagnostics (including reporting of breast MRI).
• Preparation of megablocks/large blocks and sections is recommended, as far as possible. For a more widespread use of the method, this recommendation is strong, since larger sections (sections of 4 × 6 cm or 5 × 7 cm are most common) allow for a more accurate radiopathological correlation, and a more accurate assessment of tumour size. These large blocks and slides may be prepared in pathology laboratories containing the usual infrastructure. Significantly larger sections also exist, but a special infrastructure is required in order to make them, prepare them for storage and store them. In the absence of whole slice giant blocks, digital reconstruction following scanning of sections obtained from conventional and/or mega-cassette blocks representing the entire slice may be a bypass solution. The use of large block technique is especially recommended for diffuse processes (diffuse calcification, diffusely infiltrating lobular carcinoma) and for multifocal tumours. Small (conventional) sections can only provide information of similar accuracy to large sections if they are available in large number and with complex orientation reconstruction (32), but this is much more time-consuming. In addition to large sections, it is always advisable to prepare tumour blocks of conventional size, since these allow a simpler and more economical assessment of prognostic and predictive markers by immunohistochemistry.
• With mastectomy, processing of the nipple and areola is recommended.
• For a PST, the area originally containing the tumour (optimally, clearly marked prior to treatment in a way that is visible for the pathologist), as well as its surrounding area, should be processed in detail to determine actual regression. Radiopathological comparison (specimen mammography, specimen mammography of slices) and giant block technique are recommended. Particular attention should also be paid to the detection of multifocality. If necessary, in addition to routine HE staining, cytokeratin immunohistochemistry may be used to detect residual tumour in the event of uncertainty. Comparison with a previous core needle biopsy specimen may help the assessment of regression (12). For quantifying the degree of regression, we suggest the scheme shown in Table 3 (12). The RCB (residual cancer burden) calculator, developed by the MD Anderson Cancer Center is suitable for quantification of the residual tumour volume. This calculator uses the two largest dimensions of the tumour containing tumour bed, its cellularity, including the percentage of the *in situ* carcinoma component, as well as the number of metastatic lymph nodes and the size of the largest metastasis, as variables (http://www3.mdanderson.org/app/medcalc/index.cfm?pagename=jsconvert3) (33). The advantage of this over other methods is that it strives to estimate residual tumour volume based on two dimensions and cell density, and it takes into account not only the primary tumour, but also lymph nodes (34). Pathological complete regression (pCR) can only be stated based on complete (or for large original tumours, a very thorough partial) processing of tumour bed and processing of removed lymph nodes. pCR is achieved when there is no residual invasive carcinoma in the breast and lymph nodes are also completely tumour-free: TR1 and NR1 or NR2 (35). It should be noted that for the measurement of a residual tumour in the tumour bed, the eighth edition of the AJCC Cancer staging manual sets out different principles than the guide for RCB assessment (33, 36). For the former, besides disregarding regression-induced fibrosis, the largest dimension of the largest residual tumour focus in the tumour bed is used as the basis for ypT classification (36); in the latter, the “wall to wall” distance between the most distant tumour foci in the tumour bed, with the omission of marginal fibrosis, will give the largest dimension. In the rare case, when residual tumour is found only in small vascular spaces, no primary tumor size is to be given, an this is recorded as ypT0 L1 (for the presence of lymphovascular invasion); such cases do not qualify for pCR.

**TABLE 3:** Suggestions for assessment of the regression of primary tumour (TR) and lymph node metastasis (NR) (12).

Primary tumour (TR)
1: Complete pathological regression
a: no residual carcinoma
b: no residual invasive carcinoma, but residual DCIS is present
2: Partial therapeutic response
a: minimal (<10%) residual (invasive) tumour
b: clear response to therapy but with 10–50% residual (invasive) tumour
c: clear response to therapy but with >50% residual (invasive) tumour
3: No signs of regression
**Lymph nodes (NR)**
1: No metastases, and no visible signs of regression
2: No metastases, but visible signs of regression
3: Metastasis with signs of regression
4: Metastasis without signs of regression

Lymph nodes showing multiple different therapeutic responses should be classified based on the worse response. (TR stands for primary Tumour Regression/Tumour Response, NR for Nodal Regression/Nodal Response.). (Original (i), (ii) and (iii) subcategory designations (12) have been modified to a, b and c, respectively.)

## Traditional Prognostic (Predictive) Factors

### Parameters of the Primary Tumour

One of the most important prognostic factors of breast carcinomas is the size of the invasive tumour. This should always be specified based on the largest size of the largest focus, and this is the size that determines the pT category of pTNM (Table 4) (12, 36–38). If possible, it should be measured microscopically, but for large tumours, macroscopic measurement is also acceptable. Whole tumour size, including the *in situ* carcinoma component, is important when determining locoregional treatment, so it is essential that this be specified separately. An extensive intraductal component (EIC) is usually defined as a DCIS, which accounts for >25% of the dominant invasive tumour focus and extends beyond its margins to the surrounding breast parenchyma, or as a tumour that is predominantly DCIS but contains invasive foci (39). Since such a definition of invasive tumour size and total tumour size is only obvious for unifocal tumours, tumour extent should also be specified for multifocal tumours, replacing whole tumour size; this is the largest dimension of the breast parenchyma affected by the tumour. For unifocal tumours, extent coincides with the whole tumour size. Invasive tumours may be unifocal, multifocal, and diffuse in appearance. The area between foci of multifocal invasive tumours may include tumour-free breast parenchyma, benign lesions (26, 27), or *in situ* carcinomas (27). Tumours with multiple foci of invasion can manifest in various forms: e.g., invasive carcinoma with satellite foci of invasion (the International Collaboration on Cancer Reporting (ICCR) recommends to include the size of the satellite focus and separating tumour free area in the invasive tumour size if the distance between the satellite and main tumour is less than 5 mm, and not to add the two if the distance is greater than 5 mm), EIC with multiple foci of invasion (the ICCR recommendation being to measure the largest distance between the two most distant invasive foci for invasive tumour size), multiple biologically different invasive carcinomas (considering them as two diseases if separate), cancer with extensive lymphovascular invasion (LVI; where LVI is not added to tumour size, but is part of the extent), or the tumor can be arteficially fragmented (38). Descibed scenarios may often require very individual approaches. A main feature of diffuse invasive cancers is the radiological and pathological absence of a well-defined tumour body and a spider web-like appearance (26, 27). The size of the invasive component of the tumour, whole tumour size, and tumour extent are similarly evaluated after PST, and these parameters should be determined in such cases, as well. It should be mentioned again that the AJCC recommendation for measuring the size of an invasive tumour and of lymph node metastases requires the omission of regression fibrosis when assessing tumour sizes (36), and this differs from the measurement recommended for RCB assessment (33).

**TABLE 4:** Definition of cTNM and pTNM categories for stage classification of breast cancers based on the eighth edition of the TNM (2017) (36, 37).

cT (T) and pT — primary tumour			
Pathological T category: same as clinical T classification, but only the largest dimension (rounded to the nearest mm value) of the invasive component measured on histological section will count when stating size. For larger tumours that cannot be measured microscopically in one block, the macroscopic size is also appropriate, according to the eighth edition of the TNM.			
Tx	The primary tumour cannot be assessed		
T0	No evidence of primary tumour		
Tis	Carcinoma *in situ*.		
Tis (DCIS)	Ductal carcinoma *in situ*.		
Tis (LCIS)	Lobular carcinoma *in situ* ^b^		
Tis (Paget)	Paget’s disease without associated *in situ* or invasive tumour (if Paget’s disease was associated with an *in situ* or invasive breast cancer, the latter is classified according to tumour size)		
T1	Invasive tumour of 2 cm or less in size		
T1mi	Microinvasion of 0.1 cm or less in size		
T1a	Tumour is larger than 0.1 cm, but does not exceed 0.5 cm.		
T1b	Tumour is larger than 0.5 cm, but does not exceed 1 cm		
T1c	Tumour is larger than 1 cm, but does not exceed 2 cm		
T2	Tumour is larger than 2 cm, but does not exceed 5 cm		
T3	Tumour is larger than 5 cm		
T4	Tumour of any size spreading directly to the chest wall (a) or skin (b)		
T4a	Spread to chest wall		
T4b	Oedema (“peau d’orange”) or ulceration of the skin or satellite skin nodules in the same breast		
T4c	If criteria T4a and T4b are present at the same time		
T4d	Inflammatory carcinoma (primarily a clinical staging category)		
**cN—clinical classification of regional lymph nodes** (cN and N categories are synonymous)			
cNx	Regional lymph nodes cannot be evaluated. (e.g., have been previously removed.)		
cN0	No regional lymph node metastases found		
cN1	Metastases in ipsilateral level I or II mobile lymph node(s)		
cN2	Metastases in ipsilateral fixed/conglomerate lymph node(s) or clinically detectable^a^ metastases in ipsilateral lymph node(s) adjacent to the internal mammary artery, not associated with clinically detectable^a^ axillary lymph node metastases		
cN2a	Metastases to ipsilateral surrounding structures or to (a) fixed/conglomerate lymph node(s)		
cN2b	Clinically detectable^a^ metastases in the lymph node(s) adjacent to the internal mammary artery, in the absence of clinically detectable^a^ axillary lymph node metastases		
cN3	Clinically detectable^a^ metastases in ipsilateral infraclavicular (level III axillary) lymph node(s), regardless of the involvement of level I, level II lymph nodes; or clinically detectable^a^ metastases in the lymph node(s) adjacent to the internal mammary artery and in axillary lymph node (s); or clinically detectable^a^ metastases in supraclavicular lymph node(s), regardless of the involvement of other regional lymph nodes		
cN3a	Metastases in infraclavicular lymph node(s)		
cN3b	Clinically detectable^a^ metastases in ipsilateral lymph nodes along the internal mammary artery together with 1 or more metastatic axillary lymph nodes		
cN3c	Ipsilateral supraclavicular lymph node metastases		
**pN—pathological classification of regional lymph nodes**			
At least level I dissection is required for classification and the number of lymph nodes examined should be at least 6. (TNM recommends a minimum of 6 lymph nodes, but this is for lymph node dissections and is not valid for sentinel lymph node biopsy and axillary sampling earlier performed in some United Kingdom and Scandinavian units; if there are more than 6 sentinel lymph nodes removed, the “(sn)” postscript is not applicable)			
pNx	pNx Regional lymph nodes cannot be assessed. (Not removed for examination or have been previously removed.)		
pN0	No regional lymph node metastases		
pN0(i-)	No histologically detectable regional lymph node metastases, negative IHC		
pN0 (i+)	Histologically confirmed lymph node involvement not larger than 0.2 mm or less than 200 tumour cells. (The size of the largest contiguous group of cells, if there are more groups, while in the absence of such groups the number of cells should be the criterion.)		
pN0 (mol−)	No regional lymph node metastases histologically, and negative molecular biology findings (usually RT-PCR or OSNA—one step nucleic acid amplification)		
pN0 (mol+)	No regional lymph node metastases histologically, and positive molecular biological findings (usually RT-PCR or OSNA)		
pN1mi	Micrometastasis (larger than 0.2 mm, but not larger than 2.0 mm)		
pN1	Metastases in 1–3 ipsilateral axillary lymph nodes and/or lymph nodes along the internal mammary artery; in the latter case, detected by sentinel lymph node assessment, but clinically not detectable		
pN1a	Metastases in 1–3 axillary lymph nodes		
pN1b	Metastases in the lymph nodes along the internal mammary artery, microscopic disease detected by sentinel lymph node examination only, not detectable by imaging studies or physical examination		
pN1c	Metastases in 1–3 axillary lymph nodes and in lymph nodes along the internal mammary artery, under conditions described at pN1b, for the latter		
pN2	Metastases in 4–9 axillary lymph nodes, or internal mammary lymph node metastases detected by physical examination and/or imaging, without axillary lymph node metastasis		
pN2a	Metastases in 4–9 axillary lymph nodes		
pN2b	Clinically detectable metastases along the internal mammary artery without axillary lymph node metastasis		
pN3	Metastases in 10 or more axillary lymph nodes or infraclavicular lymph nodes; or clinically detectable metastases in internal mammary lymph nodes in the presence of 1 or more metastatic axillary lymph nodes; or metastases in more than 3 axillary lymph nodes with clinically non-detectable microscopic metastases along the internal mammary artery, or ipsilateral supraclavicular lymph node metastases		
pN3a	Metastases in more than 10 axillary lymph nodes or metastases in infraclavicular lymph nodes		
pN3b	Clinically detectable metastases in lymph nodes along ipsilateral internal mammary artery with 1 or more metastatic axillary lymph nodes; or metastases in more than 3 axillary lymph nodes and in the lymph nodes along the internal mammary artery, the latter being detected only on sentinel lymph node examination, but not detectable clinically		
pN3c	Ipsilateral supraclavicular lymph node metastases.		
“pN1mi(mol+) and pN1(mol+)” Categories not accepted by the eighth edition of TNM but recommended by the European Working Group for Breast Screening Pathology and the International Collaboration for Cancer Reporting for labelling of metastases with a volume greater than pN0 (mol+), which are analysed (and thus identified almost exclusively) using quantitative molecular analysis (12,39).			
**M—distant metastases (categories cM and M are the same).**			
cM0	No distant metastases		
cM1	Evidence of distant metastasis.		
Distant metastasis is classified as pM1 only if it has undergone histological or cytological examination (i.e. metastasis has been surgically removed or sampled by biopsy); otherwise the categories are (clinical) M categories (categories Mx, pMx, pM0 are not defined).			
Stage classification			
Stage	T	*N*	M
0	Tis	N0	M0
I A	T1^c^	N0	M0
I B	T0, T1^c^	N1mi	M0
II A	T0, T1^c^	N1	M0
	T2	N0	M0
II B	T2	N1	M0
	T3	N0	M0
III A	T0, T1^c^, T2	N2	M0
	T3	N1, N2	M0
III B	T4	N0, N1, N2	M0
III C	any T	N3	M0
IV	any T	any N	M1

a Clinically detectable: structure discovered on clinical examination or imaging (excluding lymphoscintigraphy) that raises a well-founded suspicion of malignancy, or which proves to be metastatic by non-operative biopsy. The basic requirement for pN classification is pT classification after tumour removal. Consequently, if the primary tumour is not removed, only cN classification is possible, even when microscopic examination is performed on an aspiration cytology or core biopsy sample; in such cases, the suffix “(f)” refers to the microscopic examination—e.g. cN1 (f).

b The wording used in the 8th edition of the AJCC, and UICC, sources related to stages and classifications differs (36, 37). According to the former, LCIS (lobular carcinoma *in situ*) is not classified as pTis, while in the latter it belongs to pTis group.

c Including T1mi. The stages described above are those included in the TNM classification issued by the UICC, and are identical with the AJCC Cancer Staging Manual defined anatomical stages, but different from prognostic stages described in the latter source, which, in addition to ER, PR, and HER2 statuses, include grade and, when available, the recurrence score based on the Oncotype Dx test. Prognostic stages may deviate from anatomical stages by up to two subcategories in either direction (36). Dynamic changes in these prognostic stages are expected, although the provided Ref. (36) lists them on several pages, the use of online calculators could be simpler, when needed (e.g., https://reference.medscape.com/calculator/594/breast-cancer-pathological-tnm-staging).

*In situ* carcinomas can be similarly classified according to their pattern and distribution: a lesion is unifocal if it involves one single terminal ductal-lobular unit (TDLUs) or more such units located close to each other within a coherent area. An *in situ* carcinoma is multifocal (multiple) when TDLUs involved are further apart from each other and are not connected. According to Tot’s classification, an *in situ* carcinoma is considered diffuse when it primarily involves large ducts. The distribution of invasive and *in situ* carcinoma may also be summed up according to a combined pattern; if any of the components is diffuse, then the whole tumour should be interpreted as a diffuse tumour. If an invasive or *in situ* carcinoma forms multiple foci, it will be a multiple (multifocal) tumour, and it may only be considered a unifocal tumour if its invasive (and/or *in situ*) component is present in the same single focus (25, 26). Besides influencing surgical treatment, this classification also has prognostic value.

Histological type of tumours should be specified according to the WHO (World Health Organization) classification (Table 5) (40). The heterogeneous group of tumours formerly called invasive ductal carcinoma remains no special type (NST) breast cancer, suggesting that these cancers do not contain characteristics based on which they could be classified as special type cancers. The group name introduced in the 4th edition of the WHO classification was left unchanged in the 5th edition of the WHO classification (40). The classification has become significantly simpler, with a significant proportion of rare breast tumours previously classified as special tumour types now being identified as morphological variants of NST carcinomas.

**TABLE 5:** Histological classification of breast tumours according to the fifth edition of the WHO classification (40).

Tumour group	Name	ICD-0	ICD-11
EPITHELIAL TUMOURS			
Benign epithelial proliferations and precursors	Normal (typical) ductal hyperplasia		GB20.Y
	Columnar cell lesions, including atypical columnar cell transformation (FEA, flat epithelial atypia)		GB20.Y
	Atypical ductal hyperplasia (ADH)		GB20.Y
Adenosis, benign sclerosing lesions	Sclerosing adenosis		GB20.Y
	Apocrine adenoma	8401/0	2F30&XH6YZ9
	Microglandular adenosis		GB20.Y
	Radial scar/Complex sclerosing lesion		GB20.Y
Adenomas	Tubular adenoma	8211/0	2F30.0&XH7SYZ9
	Lactating adenoma	8204/0	2F30.1&XH0W31
	Ductal adenoma	8503/0	2F30.2&XH4LZ4
Epithelial-myoepithelial tumours	Pleomorphic adenoma	8940/0	2F30.Y&XH2KC1
	Adenomyoepithelioma NOS	8983/0	2F30.Y&XH2V57
	Adenomyoepithelioma with carcinoma	8983/3	2C6Y&XH7TL5
	Epithelial-myoepithelial carcinoma	8562/3	
Papillary neoplasms	Intraductal papilloma	8503/0	2F30.2&XH4LZ4
	Papillary ductal carcinoma *in situ*	8503/2	2E65.2&XH4V32
	Encapsulated papillary carcinoma	8504/2	2E65.Y&XH9XV2
	Encapsulated papillary carcinoma with invasion	8504/3	2C6Y&XH0GT6
	Solid papillary carcinoma *in situ*	8509/2	2E65.Y&XH0134
	Solid papillary carcinoma with invasion	8509/3	2C64
	Invasive papillary carcinoma	8503/3	2C60&XH8JR8
Non-invasive lobular neoplasia	Atypical lobular hyperplasia (ALH)		
	Lobular carcinoma *in situ* (LCIS), NOS	8520/2	2E65.0&XH6EH0
	Classical LCIS		
	Florid LCIS		
	Pleomorphic LCIS	8519/2	
Ductal carcinoma *in situ* (DCIS)	Intraductal breast carcinoma, NOS	8500/2	2E65.2cXH4V32
Invasive breast carcinoma	Invasive carcinoma, NST	8500/3	2C61.0&XH7KH3
	Microinvasive carcinoma		2C61.0
	Invasive lobular carcinoma	8520/3	2C61.1&XH2XR3
	Tubular carcinoma	8211/3	2C60&XH4TA4
	Cribriform carcinoma	8201/3	2C60&XH1YZ3
	Mucinous carcinoma	8480/3	2C60&XH1S75
	Mucinous cystadenocarcinoma	8470/3	2C60&XH1390
	Invasive micropapillary carcinoma	8507/3	2C60&XH9C56
	Carcinoma with apocrine differentiation	8401/3	2C61&XH4GA3
	Metaplastic carcinoma	8575/3	2C6Y&XHORD4
Rare and salivary gland type tumours	Acinic cell carcinoma	8550/3	2C60&XH3PG9
	Adenoid cystic carcinoma (ACC)	8200/3	2C60&XH4302
	Secretory carcinoma	8502/3	2C60&XH44J4
	Mucoepidermoid carcinoma	8430/3	2C60&XH1J36
	Polymorphic adenocarcinoma	8525/3	2C60&XH5SD5
	Tall cell carcinoma with reversed polarity	8509/3	2C6Y
Neuroendocrine neoplasia	Neuroendocrine tumour NOS	8240/3	2C6Y&XH9LV8
	Neuroendocrine tumour Grade 1	8240/3	
	Neuroendocrine tumour Grade 2^a^	8249/3	
	Neuroendocrine carcinoma NOS	8246/3	2C6Y&XH0U20
	Neuroendocrine carcinoma, small cell	8041/3	2C6Y&XH9SY0
	Neuroendocrine carcinoma, large cell	8013/3	2C6Y&XH0NL5
FIBROEPITHELIAL TUMOURS, HAMARTOMAS	Hamartoma		
	Fibroadenoma NOS	9010/0	2F30.5&XH9HE2
	Phyllodes tumour NOS	9020/1	
	Phyllodes tumour, benign	9020/0	2F30.3&XH50P7
	Phyllodes tumour, borderline	9020/1	2F75&XH5NK4
	Phyllodes tumour, malignant	9020/3	2C63&XH8HJ7
NIPPLE TUMOURS	Syringomatous tumour	8407/0	2F30.Y&XH9GB7
	Nipple adenoma	8506/0	2F30.Y&XH7GN3
	Paget’s disease	8540/3	2E65.5&XH3E21
MESENCHYMAL TUMOURS			
Vascular tumours	Haemangioma NOS	9120/0	2F30.Y&XH5AW4
	Angiomatosis		2E81.0Z
	Common angiomatosis		
	Capillary angiomatosis		
	Atypical vascular lesions	9126/0	
	Postradiation angiosarcoma of the breast	9120/3	2B56.2&XH6264
	Primary angiosarcoma of the breast	9120/3	2B56.2&XH6264
Fibroblastic/myofibroblastic tumours	Nodular fasciitis	8828/0	2F30.Y&XH5LM1
	Myofibroblastoma	8825/0	2F30.Y&XH8JB0
	Desmoid fibromatosis	8821/1	2F75&XH13Z3
	Inflammatory myofibroblastic tumour	8825/1	2F30.Y&XH66Z0
Peripheral nerve sheath tumour	Schwannoma NOS	9560/0	2F30.Y&XH98Z3
	Neurofibroma NOS	9540/0	2F30.Y&XH87J5
	Granular cell tumour	9580/0	2F30.Y&XH09A9
	Granular cell tumour, malignant	9580/3	
Tumours of smooth muscle origin	Leiomyoma NOS	8890/0	2F30.Y&XH4CY6
	Leiomyosarcoma NOS	8890/3	2C6Y&XH7ED4
Adipose tissue tumours	Lipoma NOS	8850/0	2F30.Y&XH1PL8
	Angiolipoma NOS	8861/0	2F30.Y&XH3C77
	Liposarcoma NOS	8850/3	2C6Y&XH2J05
Other mesenchymal tumours and tumour-like lesions	Pseudoangiomatous stromal hyperplasia		GB20.Y
HEMATOLYMPHOID TUMOURS	Lymphoma		
	MALT lymphoma	9699/3	2A85.3
	Follicular lymphoma (NOS)	9690/3	2A80.Z
	Diffuse large B-cell lymphoma NOS	9680/3	2A81.Z
	Burkitt lymphoma NOS/Acute leukaemia, Burkitt type	9687/3	2A85.6
	Anaplastic large cell lymphoma associated with breast implant	9715/3	2A90.B
MALE BREAST TUMOURS	Epithelial tumours		
	Gynaecomastia		GB22
	Carcinoma *in situ* NOS	8500/2	
	DCIS		2E65.2&XH4V32
	LCIS		2E65.0&XH6EH0
	Paget’s disease of nipple		
	Invasive carcinoma, NST	8500/3	2C61.0&XH7KH3
BREAST METASTASES			2E0Y&XA12C1
GENETIC TUMOUR SYNDROMES	*BRCA1/2*-associated hereditary breast and-ovarian cancer syndrome		2C65
	Cowden syndrome		LD2D.Y
	Ataxia-telangiectasia		4A01.31
	Li–Fraumeni syndrome, *TP53*-associated		
	Li–Fraumeni syndrome, *CHEK2*-associated		
	*CDH1*-associated breast cancer		
	*PALB2*-associated breast cancer		
	Peutz–Jeghers syndrome		LD2D.0
	Neurofibromatosis type 1		LD2D.10
	Polygenic component of breast cancer susceptibility		

a The term “neuroendocrine tumour (NET) Grade 3” is not included in the WHO publication, although the principle was to harmonize the classification of neuroendocrine neoplasms with that used for other organs. Breast NET grade is determined according to the Nottingham grading scheme, which is different from the NET grading system used for other organs; Grade 3 has not been defined. Breast NET is defined as a malignant tumour. Breast NET is rare, so the prognosis of tumours classified in this category is unknown. (Altogether, the classification of tumours into NET, NEC or NST carcinoma with neuroendocrine differentiation is somewhat controversial, these tumours require individual and multidisciplinary approaches to avoid improper management. NOS, not otherwise specified; NST, no special type.

For invasive epithelial tumours, differentiation is based on the Nottingham combined histologic grade system (Table 6) (6). For invasive tumours, the Nottingham Prognostic Index (NPI) with a proven prognostic value may also be calculated, see Table 7 (7) for help. Although prognosis of breast cancer has significantly improved since the original description, the NPI still differentiates between various prognostic groups despite better overall survival, though differences between the prognostic groups are smaller; and as an example, prognosis for the “excellent prognostic group” and the “good prognostic group” is essentially not differentiable (7). For tumours classified as pure DCIS, we also propose a three-tiered system for reporting differentiation (Table 8) (41). For the assessment of DCIS grade, there are several systems in which nuclear sizes are defined in different ways if defined at all (42); the use of these systems is not uniform, and authors of this recommendation would favour the guidelines of a consensus conference held in 1997 (42), which form the basis for German and French national recommendations (41). A commonly used prognostic factor can also be specified, the Van Nuys Prognostic Index with three variables (size, grade/necrosis, closest margin; VNPI), and its improved, upgraded version, the University of Southern California/Van Nuys Prognostic Index (USC/VNPI) including age as a fourth variable (Table 9) (43). As shown in Table 9, the Van Nuys grading is a two-component two-tiered system distinguishing between high and non-high grade nuclei and for the latter category further scoring is based on the presence or absence of necrosis.

**TABLE 6:** Combined histologic grade (Nottingham) (6).

Tissue characteristic	Points
A. Tubule formation
For the most part of the tumour (>75%)	1
To a moderate extent (10–75%)	2
To a small extent (<10%)	3
B. Nuclear pleomorphism
Small (<1.5 × normal), regular, uniform nuclei, uniform chromatin	1
Moderately larger (1.5–2 × normal) nuclei with variability in size and shape, visible nucleoli	2
Large (>2 × normal) vesicular nuclei with marked variability, multiple nucleoli	3
C. Mitotic index (depending on the size of the field of view)	See table below

		Number of mitoses in 10 high magnification fields of view		
Field of view diameter in mm	Field of view area in mm^2^	Score 1	Score 2	Score 3
0.40	0.126	≤4	5–8	≥9
0.41	0.132	≤4	5–9	≥10
0.42	0.138	≤4	5–9	≥10
0.43	0.145	≤4	5–10	≥11
0.44	0.152	≤5	6–10	≥11
0.45	0.159	≤5	6–11	≥12
0.46	0.166	≤5	6–11	≥12
0.47	0.173	≤5	6–12	≥13
0.48	0.181	≤6	7–12	≥13
0.49	0.188	≤6	7–13	≥14
0.50	0.196	≤6	7–13	≥14
0.51	0.204	≤6	7–14	≥15
0.52	0.212	≤7	8–14	≥15
0.53	0.221	≤7	8–15	≥16
0.54	0.229	≤7	8–16	≥17
0.55	0.237	≤8	9–16	≥17
0.56	0.246	≤8	9–17	≥18
0.57	0.255	≤8	9–17	≥18
0.58	0.264	≤9	10–18	≥19
0.59	0.273	≤9	10–19	≥20
0.60	0.283	≤9	10–19	≥20
0.61	0.292	≤9	10–20	≥21
0.62	0.302	≤10	11–21	≥22
0.63	0.312	≤10	11–21	≥22
0.64	0.322	≤11	12–22	≥23
0.65	0.332	≤11	12–23	≥24
0.66	0.342	≤11	12–24	≥25
0.67	0.352	≤12	13–25	≥26
0.68	0.363	≤12	13–25	≥26
0.69	0.374	≤12	13–26	≥27
0.70	0.385	≤13	14–27	≥28
**Nottingham histologic grade**				
Well differentiated, grade I	Scores 3 to 5
Moderately differentiated, grade II	Scores 6 to 7
Poorly differentiated, grade III	Scores 8 to 9

Auxiliary table for assessing the score based on mitosis index according to Chapter 6 of the European Guideline for Breast Cancer Screening (Quality assurance guidelines for pathology in mammographic screening) and the WHO tumour classification (6,40).

**TABLE 7:** Nottingham prognostic index (NPI) (7).

*No lymph nodes involved	1
1–3 lymph nodes involved	2
>3 lymph nodes involved	3
Prognostic groups based on NPI value	
Excellent prognostic group (EPG)	2–2.4
Good prognostic group (GPG)	2.41–3.4
Moderate prognostic group I (MPG-I)	3.41–4.4
Moderate prognostic group II (MPG-II)	4.41–5.4
Poor prognostic group (PPG)	5.41–6.4
Very poor prognostic group (VPPG)	> 6.41

Tumour size (cm) × 0.2 + lymph node score (according to lymph node involvement, score: 1–3*) + grade score (grade I–score 1, grade II—score 2, grade III—score 3).

**TABLE 8:** Grading of *in situ* ductal carcinomas: as recommended by the DCIS Consensus Conference (1997) (42).

Low grade DCIS (Nuclear grade 1)	Monotonous (monomorphic) nuclei with a size of 1.5–2 RBCs or of a normal ductal epithelial cell. Chromatin is usually diffuse, finely distributed, nucleoli or mitotic forms are only rarely detected. Cells are usually located in a polarized form. (The presence of nuclei of the same size but pleomorphic character will exclude low grade).
Intermediate grade DCIS (Nuclear grade 2)	Nuclei do not fall into either nuclear grade 1 or nuclear grade 3 category, they are classified as intermediate.
High grade DCIS (Nuclear grade 3)	Marked pleomorphism of nuclei with a size >2.5 RBC or of a normal ductal epithelial cell. Usually vesicular nuclei, with irregular, coarse chromatin, with visible, often multiple nucleoli. Mitosis rate may be high.

DCIS grade should be determined based on the nuclear grade. In addition, the presence and nature of necrosis (zonal/comedo or spotty), cell polarization, DCIS pattern(s) (comedo, cribriform, micropapillary, papillary, solid, other) and possible heterogeneity of grade should be reported regardless of grade.

**TABLE 9:** Assessment of DCIS prognosis: University of Southern California/Van Nuys Prognostic Index (43).

Scoring	1	2	3
Tumour size (mm)	≤15	16–40	≥41
Surgical margin (mm)	≥10	1–9	<1
Histological classification (grade)	Non-HG without necrosis	Non-HG with necrosis	HG
Age	>60	40–60	<40

With breast preservation, prognosis is good (low probability of recurrence) if the sum of scores is 4–6, moderate if it is 7–9, and poor if it is 10–12. HG: high grade (poorly differentiated). The significance of USC/VNPI, is that of an auxiliary tool for the selection of another treatment strategy after conservative surgery: cases with a high score (10–12) are candidates for mastectomy, whereas cases with a score of 7–9 for radiotherapy.

For invasive tumours, the presence or absence of peritumoral lymphovascular invasion (lymphatic and/or blood vessel invasion) should be reported.

Quantification of tumour-infiltrating lymphocytes (TIL), which can be performed on core-needle biopsy for PST, and from surgical specimens otherwise, may be a predictive and also a prognostic parameter when determining the effectiveness of (primary) systemic treatment. According to an international recommendation, only mononuclear cells/“round cells” in the stroma should be considered within the borders of the invasive tumour (Table 10) (44, 45). Based on the presence of TILs, a group of lymphocyte-predominant breast cancers (LPBC) can be distinguished (in which, in principle, there are fewer tumour cells than lymphoid stroma or lymphoid cells; this is indicated at a stromal TIL ratio higher than 50% or 60%). This type of cancer shows a higher rate of pathological complete regression after neoadjuvant treatment. TIL is mostly predictive of significant or complete regression in triple-negative and HER2-positive breast cancers (18, 46). Meta-analyses have shown that the amount of TIL is not only predictive of the effectiveness of PST (18) but also reflects the effectiveness of adjuvant treatment (47).

**TABLE 10:** Recommendation for quantification of tumour-infiltrating lymphocytes (TILs) as recommended by the International TILs/Immuno-Oncology Working Group (44,45).

0. In terms of practice, TILs can be interpreted in several localizations. Recommendation applies to a quantitative estimation of the stromal TILs (sTILs) compartment; the term TILs is used synonymously with this. The following recommendation applies to invasive breast cancers
1. The % of TILs should be expressed as the percentage of stromal area occupied by mononuclear stromal inflammatory cells (including plasma cells and lymphocytes but excluding granulocytes) as compared to the total area of the tumour stroma.
2. TILs should be assessed within the borders of the invasive tumour, which includes the invasive front of the tumour (a 1 mm zone at the tumour margin).
3. Mononuclear cells a) beyond the tumour border (invasive front), b) around DCIS, c) around normal lobules, as well as areas that d) are artificially damaged, e) are necrotic, f) show regressive hyalinization and g) showing the site of the previous core needle biopsy should be excluded from evaluation
4. Analysis of a 4–5 micron thick section per patient, examined at × 200 or ×400 magnification is sufficient.
5. Full sections should be preferred to core needle biopsies, but only the latter can be evaluated for PST
6. The average TILs should be assessed in a section, and not the most intensively infiltrated areas, exclusively
7. Quantification of TILs as a continuous variable should be performed with the highest precision possible, which in daily practice means rounding to percentages, usually ending in 5 or 0
8. It should also be considered that lymphocytes typically do not form confluent cell groups, so small empty gaps between mononuclear inflammatory cells in the TIL-infiltrated stromal area (in the numerator of the proportion; the total intratumoural stromal area being the denominator) are acceptable, and they exist even with an upper limit of 100% for stromal TILs
9. No formal limits have been set. In addition to the semi-quantitative value of stromal TILs, a descriptive name, such as “lymphocyte-predominant breast cancer” (LPBC) may also be used, in which the number of lymphocytes is basically greater than that of tumour cells; by definition, a population of lympho-plasmacytes exceeding 50% or (according to another definition) 60% of the stromal area of interest, can be identified within the tumour.

### Assessment of Axillary Lymph Node Status

Physical and ultrasound examination of the armpit is part of patients’ preoperative assessment, during which it is necessary to distinguish between patients who are clinically metastatic, i.e., node-positive (including cases confirmed by axillary ultrasound, aspiration cytology, and possibly core biopsy) and non-metastatic, i.e., node-negative patients. For this reason, targeted sampling (mostly aspiration cytology, rarely core biopsy) is part of the preoperative assessment when clinical suspicion arises. As surgical procedures change, core needle biopsy sampling is expected to become more frequent, related to (clip, magnetic or radioactive seed) marking of metastatic axillary lymph nodes before PST; however, core needle biopsy is not a prerequisite for clip insertion, since this is inserted with a separate device and may be placed after fine needle aspiration, too. In addition to establishing the diagnosis of metastasis, a sample obtained from an axillary lymph node may also be suitable for the assessment of certain prognostic/predictive factors of the tumour (ER, PR, HER2, and Ki67).

### Axillary Clearance Specimen Processing

All lymph nodes should be retrieved from the axillary fat for histological examination. Lymph nodes larger than 5 mm should be embedded, preferably cut into 2 mm thick slices, while those smaller than 5 mm should be embedded as a whole. From lymph nodes that are clearly metastatic macroscopically, embedding one single representative block is sufficient. It is advisable to choose a macroscopic slice in which extracapsular spread, if present, can also be identified. When performing the above, a methodology and marking should be used that enables reporting of the number of examined and metastatic lymph nodes at the end of the examination (e.g., staining, accurate recording of the number of lymph nodes per block if more than one lymph node is included in a block).

For axillary lymph nodes removed after PST, knowledge of the pre-treatment lymph node status and communication of this to the pathologist is essential. In addition to lymph nodes, small connective tissue masses, which are often only palpable, should also be examined. Routine use of cytokeratin immunohistochemistry in patients with lesions that suggest only scarring and regression is not warranted; however, for an HE finding suggestive of a tumour, it may help to assess the presence of residual tumour.

### Sentinel Lymph Node

• For pathologists, a lymph node sent by a surgeon with such designation is considered a sentinel lymph node.
• Basic examination of sentinel lymph nodes is embedded histological examination.
• Broadly speaking, sentinel lymph node involvement by micrometastases (see TNM staging in Table 4) or otherwise occult metastases that can be detected only by using special techniques, have minimal prognostic value (48). Short-term results from surgical randomized studies of micrometastases do not support completion axillary lymph node dissection for such cases (49, 50), and according to international recommendations, systemic treatments are never based solely on the presence of micrometastases (47, 48). Therefore, it appears that there is no need for a processing of sentinel lymph nodes that is more thorough than the one suitable for the detection or exclusion of metastases larger than micrometastases (i.e., macrometastases). As a first approach, a negative sentinel lymph node sent to the pathology department should be processed in a way that allows to rule out the presence of macrometastases as reliably as possible. For this, it is sufficient to examine the HE-stained section of slices made in 2 mm increments. When needed (e.g., for uncertain HE finding of lobular carcinoma or for suspected malignant cells after PST), cytokeratin immunohistochemistry may be used as a complementary method. After PST, minimal residual tumour (even the presence of isolated tumour cells) will indicate axillary lymph node dissection (20), but the recommendations do not consider more extensive processing and routine immunohistochemistry necessary even in this setting (12). In the first approach, for metastatic lymph nodes, a minimal examination providing the most accurate information about the metastasis (e.g., histological examination of the section representing the largest dimension) will be sufficient.
• Pathological processing of sentinel lymph nodes can be tailored based on clinical picture and need: if axillary lymph node dissection is not planned in the first instance for patients with clinically negative axillary status in cases of sentinel lymph node involvement (51–54), then intraoperative examination is not useful. In other cases, intraoperative evaluation may also be required. The aim is to detect right away as many of the metastatic sentinel lymph nodes as possible, so that any axillary clearance that becomes necessary can be performed in one operative session, if possible. However, it should also be taken into account that intraoperative microscopic examinations are not able to identify all metastases; their sensitivity is low, especially for micrometastases. Both cytology and intraoperative frozen section histology are suitable for intraoperative examinations, but frozen serial sectioning of the entire lymph node is contraindicated. Based on a meta-analysis, the sensitivity of a frozen sections is approximately 10% higher than that of imprint cytology (55, 56). Validated assays based on quantitative reverse transcription polymerase chain reaction or loop mediated isothermal amplification are also suitable for intraoperative examination of metastases. (Most of these have been calibrated so that cases falling into the “isolated tumour cell” category are not classified as metastatic.) As a basic principle, a lymph node should not be used in its entirety for a poorer quality intraoperative examination.

### Special Assessment of Prognostic and Predictive Factors (Steroid Hormone Receptors and HER2 Determination, Ki67)

The factors listed in this subheading are items that currently influence the treatment of breast cancer and need to be examined separately.

• Fixation of the fresh specimen should start as soon as possible: immediately or, for optimal receptor determination, no later than 30–60 min after excision, in 10% formalin kept in a refrigerator at 4°C, in a minimum of 5 times the volume of the specimen (57). If the material is not delivered to the pathology department within 2 h, it is advisable to store it in the fixative solution in a refrigerator at 4°C until delivery, with uniform formalin penetration, fixation without crusting, ensuring the best preservation of proteins (even phosphorylated potential signal path targets), and nucleic acids (58, 59). If the fresh sample cannot be delivered from the surgical to the pathology department within an optimal time limit (maximum 60 min), vacuum packaging and storage at 4°C, followed by delivery within up to 16 h is a validated alternative (60). Efforts should in any case be made to refrigerate the fresh sample to 4°C and deliver it as such, since this takes priority over transport at room temperature or higher, with or without formalin (and regardless of vacuum packaging) (58, 59). Duration of fixation for core biopsies is a minimum of 6 h; for surgical specimens, in the case of 5–10 mm thick slices prepared before fixation, an optimal duration of 24 h and up to 72 h is recommended (57, 61). For optimal receptor assessment, sections prepared on adhesive slides as freshly as possible within a maximum of 3 days are recommended. If the immunostains are performed later, fresh sections may be stored at 4°C in a dark place, away from air as much as possible (e.g., in a slide storage box, in contact with each other) for at least 2 months without significant antigen/DNA loss, and it is therefore recommended that control sections are stored in the same way (62).
• If predictive and prognostic factors need to be assessed from a metastasis (body cavity fluid) or, in the absence of other specimen, from a fine needle aspiration sample, only a formalin-fixed smear or cell block may be used for HER2 immunohistochemistry to avoid the high false positivity that occurs with alcohol fixation (63, 64). In the assessment of prognostic and predictive factors from cytological samples, the highest concordance with histological samples was shown for formalin-fixed, paraffin-embedded cell blocks, so efforts should be made to use this. For a cell block, the pre-analytical phase should be standardized similarly to tissue techniques. Whenever possible, whether for fine needle aspirate or a body cavity fluid, samples should be fixed in 10% buffered formalin for a minimum of 6 h and a maximum of 48 h. Afterwards, the cell block method used should be followed, and then the cell block should be treated similarly to the histological specimen (65–68). Using cell block techniques, predictive markers can be reliably assessed under conditions similar to histological specimens (69).
The optimal method for steroid hormone receptor determination is immunohistochemistry. Laboratories examining prognostic and predictive markers using immunohistochemistry are expected to participate in an external quality control programme and achieve appropriate qualification for their performance, with particular emphasis on samples sent by the quality control centre. In the context of steroid hormone receptor (oestrogen and progesterone receptors, as well as androgen receptors) testing, “oestrogen receptor” (ER) usually refers to the alpha subtype. There is still insufficient prognostic or predictive experience with oestrogen receptor beta and androgen receptors (AR) to require their assessment, although AR may be requested for triple negative tumours. Tumours with a staining rate of 1% or more are considered positive (10), although there is no doubt that tumours with staining between 1 and 10% have lower hormone sensitivity (69, 70). In light of these, the estimated proportion of positive cells and the average intensity of staining should be specified in the report. Cases showing no staining and those with less than 1% staining are considered hormone receptor negative. According to the latest recommendation, cases with an ER positivity of ≥1 and ≤10% should be classified into a new diagnostic category of “low positive/weakly positive” [The low positive designation applies only to invasive carcinoma and ER, and is not used for PR or DCIS (71, 72)]. In such cases, the result may warrant additional steps (re-testing of controls, involvement of a second examiner, validated digital quantification, comparison with previous samples taken from the patient, re-testing on the same or an alternative block) and require additional comments. These comments could include for example: “The cancer in this sample has a low level (1–10%) of ER expression by IHC. There are limited data on the overall benefit of endocrine therapies for patients with low level (1–10%) ER expression but they currently suggest possible benefit, so patients are considered eligible for endocrine treatment.” There are data that suggest invasive cancers with these results are heterogeneous in both behavior and biology and often have gene expression profiles more similar to ER negative cancers. In the absence of internal tissue control (and only if the external tissue control is adequate), it may be mentioned that ER status could be more reliably verified on a sample containing internal tissue control, if required (71). A more accurate prediction of therapeutic effects is provided by the semi-quantitative rapid scoring system proposed below (Allred quick scoring (4); Table 11) (To avoid false negativity, it is advisable to choose a block that also has a non-tumorous epithelial element as an internal control. In its absence, or if based on the histological type or grade, a negative reaction is unlikely, it is recommended that it be repeated with adequate controls). Antibodies with IVD (*in vitro* diagnostic) labelling are preferred for assessment. Examination of a large number of samples in external quality assurance programmes (UK NEQAS and NordiQC) has shown that false negativity is mainly due to insufficient antigen retrieval (over-fixing), so in doubtful cases it is advisable to increase the epitope retrieval time by approximately 30%–50% (73).
• In practice, assessment of HER2 status is justified for invasive cancers; the test is based partly on the degree of HER2 protein over-expression (immunohistochemistry, IHC) and partly on HER2 gene amplification (*in situ* hybridization, ISH). A practical cost-effective approach, in line with international recommendations, is that samples evaluated as 3+ on immunohistochemistry represent a positivity that allows for targeted anti-HER2 treatment. To avoid false positivity in 3+ cases, where the histological type or grade contradicts this HER2 status [tubular carcinoma, mucinous carcinoma, grade I no special type (ductal) carcinoma], it is recommended to repeat at least the HER2-IHC reaction. Samples rated 2+ by immunohistochemistry require further molecular testing, while samples rated 0 or 1+ based on HER2 immuno-staining, are considered negative for targeted treatment and prognosis. If classification based on immunohistochemical reaction is uncertain, an ISH test is justified. Rules and algorithm for determining HER2 status are shown in Table 12 (14, 74–76).
• Of the HER2-ISH assays, fluorescence *in situ* hybridization (FISH) is the most widely used. For the evaluation of tumours with inconclusive results on IHC or FISH, the American Society of Clinical Oncology/College of American Pathologists has formulated 3 groups and recommendations for evaluation (Table 12) (14, 61). A suitable alternative to FISH can be the chromogenic (CISH) or the silver-enhanced (SISH) method. A combined method approved by the Food and Drug Administration for the combined assessment of HER2 amplification with chromogenic ISH (dual colour ISH, DISH) and of protein-level HER2 expression (IHC) is also available in the United States. This assay, known as GPA (gene-protein assay), may yield discordant results in some cells (77). IHC results seem to better reflect the efficacy of anti-HER2 treatment (78).
• More recently, clinical trials testing novel targeted drugs for breast cancers demonstrating a low level of HER2 expression/amplification require reconsideration of the HER2-negative vs. HER2 positive dichotomization. A category of HER2-low has been introduced for cases demonstrating IHC scores 1+ or 2+ without ISH evidence of amplification (76).
In addition to the mitosis rate, IHC testing of the Ki67 proliferation marker is the most common way of assessing proliferation. In such cases, the percentage of positive tumour cell nuclei relative to the total number of tumour cells should be reported, regardless of the intensity of the reaction. There are several suggestions and recommendations for quantification, as well as for limits serving to distinguish between high and low proliferation tumours. Until there are internationally accepted long-term recommendations, we recommend using an estimate with a 5% accuracy, when assessing the Ki67 labelling index for breast cancers. According to the 2015 St. Gallen recommendation on Ki67 labelling (69), cases of high and low proliferation are not separated by a cut-off point, but there is a value below which proliferation is clearly low (approximately 5%–10%) and there is a value above which it should be considered high (approximately 25%–30%), while in the zone between them, the Ki67 labelling index is interpreted as uncertain. At the latest, 2021 St Gallen consensus meeting, a majority of panellists (62%) agreed with the statement of the International Ki-67 Working Group that in women with ER-positive HER2-negative T1–2 N0–1 breast cancer a low Ki-67 ≤5% would not warrant chemotherapy, whereas a Ki-67 ≥30% would justify chemotherapy. In node-negative ER-positive PR-positive HER2-negative tumours, the majority (42%) voted for a Ki-67 of at least 30% for recommending chemotherapy. It should be noted that 36% of the panel members stated the threshold is not known. In ER-positive HER2-negative breast cancer, Ki-67 should be tested in all cases according to 61% of the panel, while 30% would only order Ki67 if chemotherapy is considered and a genomic signature is not available (79, 80). The Ki67 zone, which determines low and high proliferation, may be different for different implications (e.g., as an indication of adjuvant treatment, expected efficacy of neoadjuvant treatment, or estimation of actual efficacy as measured by interim core biopsies). If there is any doubt, a Ki67 reaction performed on a paraffin-embedded tonsil section fixed for 72 h (with external quality assessment granted) may demonstrate the suitability of the method and serve as a control (if there is uniform positivity of dark zone B cells in germinal centres and positivity in every 5th to 10th basal cell layer or every 2nd to 3rd supra-basal cell layer cell in the epithelium). Although Ki67 is one of the recommended prognostic factors, its assessment may be skipped if there is a high mitosis rate (e.g., twice the mitosis score required for mitosis score 3, when grading).
• In some tumours (thus far only triple-negative, metastatic breast cancers), assessment of PD-L1 has become widespread, and testing was recommended to be performed in the metastatic tumour, if possible. Based on evidence from a clinical trial (81), although SP142 is the antibody with the weakest performance among anti-PD-L1 antibodies tested (82), PD-L1 positivity determined by it may be an indicator of the efficacy of immunotherapy (atezolizumab) and it is currently a prerequisite for this treatment. The reaction can be performed on a (core) biopsy or on surgical material. Due to the need for a costly infrastructure, this testing is only possible when there is an oncological indication and assessment is done in a few breast centres and not all countries. It cannot be done routinely yet. Positivity by IHC has a defined set of criteria, which for a tumour to be considered positive mainly requires that the proportion of the area occupied by PD-L1-positive “immune cells” in the evaluable stroma of the tumour is equal to or greater than 1%. Although we maintain the text relating to atezolizumab related PD-L1 testing, it must be mentioned that the United States Food and Drug Administration has suspended the accelerated approval of atezolizumab for metastatic triple negative breast cancer, and accordingly the National Cancer Collaborative Network (NCCN) guideline has removed the footnote advising testing for PD-L1 for the identification of candidates for atezolizumab therapy (83). At the time of writing, no related European Medicines Agency action has been noted, and atezolizumab is still a treatment option in Europe. Another clinical trial evidence supports the addition of pembrolizumab to chemotherapy in metastatic triple-negative breast carcinoma, but the biomarker test here involves the 22c3 antibody and a CPS (combined positive score) of 10 or above (84).

**TABLE 11:** Assessment of oestrogen and progesterone receptors by Allred quick scoring (QS) system (4).

Average intensity	Points
Negative	0
Weak	1
Intermediate	2
Strong	3
**Proportion of positive nuclei**	**Points**
No	0
<1%	1
1–10%	2
10%–1/3	3
1/3–2/3	4
>2/3	5

The sum of the two subscores will give the total score. Possible values: 0, 2–8. (Response to endocrine therapy is expected for a score >2, and the response is expected to increase proportionally with the score). In theory, ER (PR) status can be Allred+ (Allred QS > 2) with <1% staining (<1% 2+, Allred QS 3 or <1% 3+, Allred QS 4), these are interpreted as negative. If recurrent or metastatic tumours are examined, steroid hormone receptor assessment should be repeated. Pathology departments performing predictive immunohistochemical tests are expected to participate in an external quality assurance programme and achieve appropriate qualification. The use of an external control tissue is recommended, and it is advisable to select a block for the immunohistochemical reaction that includes an internal control.

**TABLE 12:** Assessment of HER2 tests^a^ (14, 74–76).

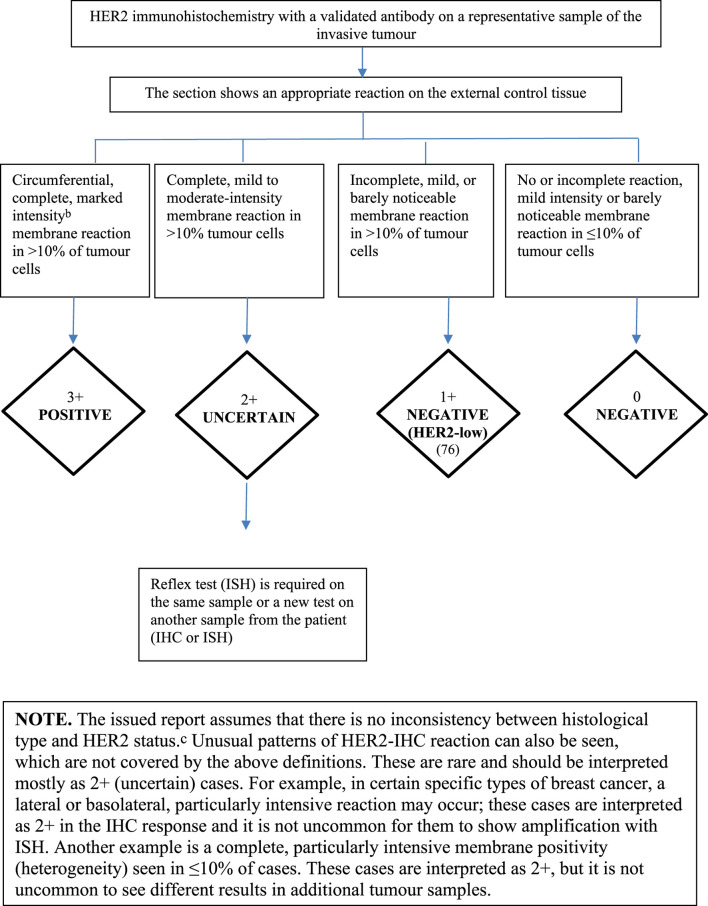				
**Grouping based on HER2 (dual probe) ISH result** 1. Group 1: POSITIVE, *HER2*/CEP17 ratio ≥2.0 AND mean *HER2* copy number per cell ≥4.0				
2. Group 2: *HER2*/CEP17 ratio ≥2.0 AND mean *HER2* copy number per cell <4.0. Considered positive only if IHC is 3+				
3. Group 3: *HER2*/CEP17 ratio per cell <2.0 AND mean *HER2* copy number ≥6.0. Considered positive only if IHC is 2+ or 3+				
4. Group 4: *HER2*/CEP17 ratio <2.0 AND mean *HER2* copy number per cell ≥4.0 but <6.0. Considered positive only if IHC is 3+				
5. Group 5: NEGATIVE, *HER2*/CEP17 ratio <2.0 AND mean *HER2* copy number per cell <4.0				
**ISH groups**	**Biology**	**HER2/CEP17 ratio**	**Mean HER2 copy number**	**2018 ASCO/CAP recommendation**
1	Classical HER2-amplified tumour	≥2	≥4	Positive
2	Chromosome 17 monosomy	≥2	<4	Negative (HER2-low if IHC 1+/2+; 76) unless HER2 IHC is 3+^d^
3	Co-amplification (previously chromosome 17 polysomy)	<2	≥6	Negative (HER2-low if IHC 1+; 76); unless HER2 is IHC 2+ or 3+
4	Borderline/uncertain	<2	≥4 and <6	Negative (HER2-low, if IHC 1+/2+; 76) unless HER2 is IHC 3+
5	Classical HER2 non-amplified tumour	<2	<4	Negative (HER2-low, if IHC 1+/2+ (76)
Summary of ASCO/CAP HER2 Professional Recommendation of 2018.				
Cases rated 3+ are considered positive for targeted treatment, while those rated 2+ are considered uncertain, including cases showing strong membrane staining in <10% of cells. Cases rated 0 and 1+ should be considered negative. (F)ISH: this is mandatory in cases of uncertain HER2 status with IHC.				
HER2-low category encompasses non-amplified IHC 1+ and 2+ cases, and accordingly the “non-positive” cases of ISH groups 2, 3 and 4 (76).				
^a^Based on the latest (2018) ASCO/CAP recommendations (ASCO/CAP).				
^b^Clearly visible at low magnification in a homogeneous, contiguous tumour cell population.				
^c^HER2 positivity is virtually non-existent in the following tumour types:				
Histological grade 1 NST carcinomas Classical lobular carcinoma, oestrogen and progesterone receptor positive Tubular carcinoma Mucinous carcinoma Cribriform carcinoma Adenoid cystic carcinoma^d^In the case of HER2 monosomy, there is clinical evidence, based on retrospective analysis, that these may respond to targeted treatment in the same way as HER2 positive tumours, suggesting that targeted treatment should be considered for this group (75). HER2 testing should be performed on the surgical specimen in the following cases, even if this has previously been done on the core biopsy specimen: if the core biopsy sample contained a small amount of tumour tissue or the invasive component of the tumour was visible only in the surgical specimen. if the surgical specimen shows a high grade carcinoma not seen in the core biopsy specimen, or morphological heterogeneity or a different additional tumour nodule that was not represented by the core biopsy (30). if it is suspected that a preanalytical error has occurred during the processing of the core biopsy sample. if the HER2 assessment in the core biopsy sample yielded an uncertain result if HER2 positivity in the core biopsy sample was heterogeneous in a tumour remaining after neoadjuvant treatment.For recurrent or metastatic tumours, HER2 assessment should be repeated. **Heterogeneity of HER2**Definition of heterogeneity: an aggregated cell population consisting of amplified cells that make up >10% of tumour cells in the section examined. Individual amplified cells present in a mosaic-like, scattered distribution do not fall into this category. Cases as defined above are rare. Amplified and non-amplified areas should be examined separately, and *HER2* / CEP17 ratio and mean *HER2* copy number per cell in the two cell populations should be reported separately. The proportion of the amplified tumour cell population should be specified in the report. Cases with non-amplified and amplified areas should be considered HER2-positive. In the event of morphological heterogeneity, it is recommended to repeat HER2 testing on the surgical material (74). Pathology departments performing predictive immunohistochemical tests are expected to participate in an external quality assurance programme and achieve appropriate qualification.				

### The Histopathology Report

Histopathology reporting of breast cancer can be done in a free text format, but it is recommended that a standard form be used, containing information about each of the essential elements (38). As an important part of the report, clinically relevant prognostic factors that can be assessed during the pathological examination should be specified. A short and clinically oriented summary of these factors is recommended, in accordance with the attached sample report. The range of relevant and independent prognostic factors, as well as predictive factors that are critical in terms of the treatment specified in the sample (Sample histopathology report), is currently considered sufficient. Other factors are either not sufficiently significant (e.g., necrosis, elastosis, etc.) or their independent prognostic value has not thus far been demonstrated (e.g., perineural invasion, ploidy, telomerase, cathepsin D, etc.). It should be noted that the Nottingham combined histological grade may also carry, with a few rare exceptions (e.g., adenoid cystic or mucoepidermoid carcinoma), prognostic information in more common special type breast cancers, so the use of grading is also recommended in the latter cases. A summary of the cytology report content is also provided along with the histopathology sample report.

## Multigene Molecular Tests

Over the last 2 decades, multigene tests based on molecular techniques have become more widespread. These may help in determining the nature of the oncological treatment to give (most often the need for chemotherapy or whether this can be omitted), or may be an indirect reference for choosing therapies by classifying tumours into molecular subtypes, and giving information on prognosis (recurrence). These commercially/provider-available tests examining the expression profile of specific genes are expensive, and only some of them are available with public funding, based on the recommendation of an oncology team. In some cases, when the indication for chemotherapy cannot be determined based on the conventional prognostic and predictive factors detailed above, such a test may be warranted. According to evidence resulting from the prospective randomized trial (TAILORx), OncotypeDx, based on the examination of the expression of 21 genes, is not only prognostic but also predictive of the efficacy of chemotherapy in ER+ HER2− pN0 breast cancers, and in general a recurrence score (RS) can be specified with which chemotherapy complementing endocrine therapy is not expected to have a significant effect, or above which chemotherapy has a survival benefit (85). The RxPONDER trial suggests that the same RS limit (25 or lower) identifies postmenopausal women with breast cancer who do not benefit from the addition of chemotherapy to endocrine treatment (86). Another test, EndoPredict, may be suitable for assessment of the efficacy of chemoendocrine therapy, based on a retrospective comparative study providing more limited evidence (87). A prospective randomized trial (MINDACT) evaluating the value of prognostic information provided by MammaPrint, a test based on examination of expression of 70 genes, concluded that among patients for whom risk assessment based on clinical and conventional pathological factors and gene expression led to contradictory results, genomic testing makes sense only in patients with clinically high risk. In some (nearly half) of these patients, chemotherapy can apparently be omitted based on a low genomic risk (88). In addition to the above, there are other studies on multigene prognoticators, of which the prognostic results are extrapolated to assess the presumed efficacy of chemotherapy administered in addition to endocrine therapy. The Prosigna (PAM50) test provides not only a molecular, gene expression profile-based classification of the tumor (luminal A, luminal B, HER2-enriched, basal like), but also provides a risk of recurrence (ROR) score, this may help in estimating the prognosis (89). Since this is a dynamically developing applied discipline, recommendations may change over time; it is most appropriate to choose the test in the light of existing evidence and clinical questions.

In addition to the above multigene, predominantly RNA-based assays, targeted therapies for breast cancer may require the assessment of additional DNA-based tests for gene mutations. Currently, germline BRCA1-2 mutation testing is the most common investigation for PARP (poly-ADP ribose polymerase) inhibitor treatment. Since this mutation analysis for breast cancer is performed on blood samples, a clinical geneticist should evaluate the results, and genetic counselling is required. Testing for gene mutations responsible for resistance to endocrine or CDK4/6 (cyclin-dependent kinase 4/6) inhibitor therapy either from tumour tissue or free circulating tumour DNA isolated from plasma are another group of multigene tests. Common guidelines for testing for these mutations have not yet been developed. Molecular tests are performed in specialized laboratories; our most important task is to maintain the quality of the sample by optimal fixation and processing conditions. This is particularly important in view of the fact that prognostic multigene tests are RNA-based, and RNA is more vulnerable than DNA. It is recommended that a multidisciplinary team decides whether these tests are to be run. Table 13 provides a brief overview of the currently most widely used multigene tests and examinations of hotspot mutations required for targeted therapies (85–93).

**TABLE 13:** Overview of multigene expression-based/molecular prognostic tests (85–93).

Test	Methods	Number of genes/proteins tested	Role of patient group/test	ASCO/NCCN recommendation
OncotypeDX Tumour RNA	RT-PCR	21 genes (16 genes + 5 references genes)	ER/PR+, HER2-, pN0 ER/PR+, HER2-, pN1/Estimation of the recurrence risk, assessment of the need for chemotherapy (predictive and prognostic)	strong
MammaPrint Tumour RNA	Microarray	70 genes	ER/PR+, HER2-, pN0 ER/PR+, HER2-, pN1/Estimation of the recurrence risk, assessment of the need for chemotherapy (prognostic)	strong
Prosigna (PAM50) Tumour RNA	Microarray	50 genes + 5 references genes	ER/PR+, HER2-, pN0	intermediate
EndoPredict Tumour RNA	RT-PCR	12 genes (8 genes + 3 RNA references genes + 1 DNA references gene)	ER/PR+, HER2-, pN0 Assessing the need for chemotherapy, prolonged hormone therapy	intermediate
Germ cell mutation testing Non-tumour-derived DNA from blood	Sanger sequencing or NGS	BRCA1-2	Screening for hereditary breast cancer: Patients under the age of 40 years, significant family history of breast cancer, triple-negative breast carcinoma, history of ovarian cancer, susceptibility to PARP inhibitor therapy	strong
Gene panel test: hotspot mutations, amplifications, fusions; microsatellite instability (tumour DNA, RNA)	NGS, PCR, FISH, IHC	ESR1, PIK3CA, RB1, FGFR1, NTRK, microsatellite markers, MLH1, MSH2, MSH6, PMS2	Hormone therapy resistance, CDK4/6 inhibitor resistance…	Indication depending on clinical picture

Multigene testing methods (comprehensive genomic analysis) in which a potential resistance mechanism and/or therapeutic target is sought based on tumour-specific abnormalities may also be used, although these methods are used rarely because of the versatility of therapeutic options in breast cancer (94). Multidisciplinary decision-making is also crucial in this respect. In rare cases, molecular testing may also be performed to support a diagnosis (e.g., detection of ETV6-NTRK3 translocation typical of secretory carcinoma).

Use of tissue markers (the old-fashioned method to insert foreign tissues, generally from cadavers or benign surgeries, for either identification or orientation purposes) endangers the effectiveness of molecular tests, and therefore this traditional way of identifying and orienting the sample “in the 21st century era of targeted molecular diagnostics and modern patient rights, is a completely obsolete and unacceptable practice and should therefore be abandoned” (95).

Liquid biopsies are suitable for targeting circulating tumor cells (CTC) or circulating tumor DNA (ctDNA). Fields of application include 1) initial detection of oncogenic and targetable mutations, 2) response monitoring: under successful therapy, decrease of cell-free DNA (cfDNA) and ctDNA levels in blood; 3) identification of (actionable) resistance mutations in patients under therapy. One of the possible mechanisms for resistance in ER+HER2-cancers might be due to the dysregulation of phosphoinositide 3 kinase (PI3K)/Akt/mammalian target of rapamycin (mTOR) signalling pathway (96). Using blood components for liquid biopsies has become important in assessing *PIK3CA* mutations in ctDNA in breast cancer patients. Multiple techniques have been employed to isolate and analyse breast cancer ctDNA with high sensitivity and specificity (97). Without additional invasive testing, analysis of ctDNA in metastatic breast cancer for the presence of PIK3CA mutations have been successfully used in clinical oncology. Of several methodologies employed for *PIK3CA* mutation detection from liquid biopsies, digital droplet PCR has been proposed as the most sensitive approach which can detect mutations in ctDNA even in the non-metastatic setting (98). This allows timely follow-up, potentially overcoming spatial and temporal heterogeneity of tumour. Liquid biopsies can be analyzed in different settings, including pathology laboratories.

## Immunophenotype—“Surrogate” Tumour Types

Since molecular subtypes of breast cancer were first described, there has been a growing need for pathologists to classify tumours, based on the pattern of immunohistochemical stains used in the everyday diagnosis of breast cancer, into surrogate subtypes that approximately reflect molecular subtypes. According to the recommendations of the St. Gallen Consensus Conference in 2015 (69), triple-negative and HER2 groups are well defined among oestrogen receptor negative tumours, along with the luminal A-like oestrogen receptor positive cancers. But a significant group of hormone receptor positive tumours (called “luminal B-like”) is very heterogeneous and difficult to define. The latter group includes tumours with low steroid hormone receptor expression, increased proliferation, and/or concomitant HER2 positivity. The 2013 and 2015 St. Gallen recommendations form the basis for this classification (69, 70), which is shown in Table 14; the content of the table has been valid since then. However, it should be noted that with proper definition of what each surrogate subtype means, it is not a mistake to simply describes the tumour in question with the phenotype (e.g., ER+ HER2+), as this will be understood. However, it is not recommended to classify as “luminal A” any tumour that, according to IHC, appears to be luminal A-like. Luminal A, luminal B, basal-like, “HER2 enriched” types are based on a gene expression profile; in addition to their definition, a prognosis-related score (ROR, risk of recurrence) can also be given.

**TABLE 14:** Immunohistochemistry classification for therapeutic classification of breast cancers based on the recommendations of the St. Gallen Consensus Conference of 2015 (69).

Clinical classification	Notes
Triple negative	ER−/PR−/HER2−
Hormone receptor negative, HER2-positive	See criteria above
Hormone receptor positive, HER2-positive	See criteria above
Hormone receptor positive, HER2-negative: spectrum of luminal tumours	
Strong hormone receptor positivity, low proliferation, low tumour mass (luminal A-like)	Strong hormone receptor expression, low Ki67 labelling index. pN0-pN1, pT1-pT2
Intermediate	
Less hormone receptor positive, increased proliferation, high tumour mass (luminal B-like)	Lower hormone receptor expression, high Ki67 labelling index, ≥pN2, histological grade 3, extensive lymphovascular invasion, ≥pT3

Notes. ER positivity between 1% and 9% was considered uncertain by the St. Gallen consensus conference, rare tumors with this range of positivity have generally worse prognosis than those with higher range of ER positivity. The assessment of the Ki67 labelling index should be based on the average Ki67 values of each laboratory: e.g., if the median Ki67 labelling index is 20%, then a value below 10% is clearly low, a value of 30% or above is certainly high. As an update to this approach, the 2021 StGallen/Vienna Consensus proposed values >30% as an indication for chemotherapy in ER-positive tumours (79).

## Clinical Trials—Role and Duties of the Pathologist

With the acceleration of targeted drug development, more and more patients are being treated in clinical trials, in which tumours are most often re-examined, or a target molecule or biomarker needed for treatment is assessed in a central laboratory. In such cases, cooperation with the pathologist diagnosing the tumour is required. A prerequisite for cooperation is that the pathologist is involved in the clinical trial, as the specialist creating the report serving as the basis for enrolment; as such, they should be informed of the details and objectives of the trial and their participation should be part of the contract. Preferably, the pathology department should be contracted by the study sponsors, to inform the participating pathologists about trial goals and material requirements as well as to ensure proper reimbursement of trial-related procedures. The specimen specified in the protocol must be released by the pathologist under the specified conditions and the delivery/dispatch of the block (or the requested specimen) should be documented. A similar situation may arise with regard to sample selection for multigene expression tests. For a limited amount of tumour tissue, division of the sample should also be considered.

## The Pathologists’ Role in the Multidisciplinary Team

The diagnosis and treatment of breast cancer is a multidisciplinary collaboration between different medical and paramedical professionals. As mentioned before, the diagnosis of breast cancer and its differential diagnosis requires radiopathological and clinicopatholgical correlation. Adjuvant, neoadjuvant and palliative therapy related decisions are founded on prognostic and predictive markers, identified target molecules determined by pathologists. The interpretation of these results is not always straight forward, and communication by solely reports may lead to misunderstanding and harm to the patient. This is why it is expected that pathologists present their findings at the multidisciplinary tumor boards, interpret any limitations and take part in the decision-making process.

## Conclusion—Objectives to Be Achieved in the Future

As a conclusion to the text on pathology, here are some of the recommendations proposed by the expert panel, the implementation of which requires policy support, but which may contribute to a higher standard and better quality of professional practice, performed under better circumstances.

In the recommendations above, quality assurance is mentioned in two aspects, namely: an endeavour for cytology laboratories establishing the diagnosis; and a requirement for pathology laboratories involved in predictive immunohistochemistry. In the future, it seems to be a realistic goal that all pathology units involved in the screening and diagnosis of breast cancer should certify their professional competence using external quality control. Generally speaking, however, pathology laboratories should be prepared to achieve a higher level of quality, the elements of which are included in the requirements of ISO 15189 (99). The new *in-vitro* diagnostic regulation (Regulation (EU) 2017/746; IVDR) will come into full effect after a transition period ending in May 2027. This EU regulation replaces the directive 98/79/EC of the European Parliament on *in vitro* diagnostic medical devices (IVDD). The implementation of the IVDR has significant impact on medical laboratories, including pathology laboratories. Accordingly, laboratories will have to be accredited according to standards ISO 9001 or 15189.

• In addition to the technological external quality control indicated above, there is justification for setting up a centrally organized diagnostic (and reporting) programme for pathological units involved in breast cancer screening and diagnosis, in order to improve and ensure compliance, with the necessary infrastructure and financial resources.
• It would be appropriate to install specimen mammography devices in high throughput breast diagnostic pathology departments (the EUSOMA recommendation of 150 cases/year may be relevant here, see under “Non-operative diagnostics (preoperative or pretreatment biopsy diagnosis)”.
• In line with the panel of radiology experts, we recommend that if an expert is involved in the diagnosis or false diagnosis of breast cancer in case of suspected error (e.g., legal dispute, claim for compensation, etc.), the expert should be a person with documentable experience in this field. Non-pathologists and general pathologists who examine small numbers (<100 per year) of cases and have no experience in evaluating samples obtained from screening should not be accepted as experts. In order to give an opinion, an expert must simulate a real-life situation (they should not analyse the appropriateness of preoperative diagnosis and therapeutic decision retrospectively, with the knowledge of the detailed results of all investigations and surgical-histological reports). It is recommended that the expert form an opinion only on the basis of the information available at the time of the decision(s) contested in the dispute/lawsuit, evaluating the case in question together with several similar, anonymised cases.
• Development and investment in the field of digital pathology are also necessary. The possibilities of these developments are multifold and include teaching, quality control, consultation, morphometry, image analysis; and digital material is the *sine qua non* of artificial intelligence-based diagnostic, predictive algorithms.

This is part 2 of a series of 6 publications on the 1st Central-Eastern European Professional Consensus Statements on Breast Cancer covering imaging diagnosis and screening (100), pathological diagnosis (present paper), surgical treatment (101), systemic treatment (102), radiotherapy (103) of the disease and related follow-up, rehabilitation and psycho-oncological issues (104).

## Author’s Note

The consensus document contains product placement without the intention of advertising. Each complex molecular test is unique, and although these can be described without indicating their name (for example with the number of genes tested), not everyone will necessarily understand what this refers to. For this reason, and adopting the practice used in some of the source works, the tests are listed under their trade name.
